# Influence of Schumann Range Electromagnetic Fields on Components of Plant Redox Metabolism in Wheat and Peas

**DOI:** 10.3390/plants11151955

**Published:** 2022-07-27

**Authors:** Natalia Mshenskaya, Yulia Sinitsyna, Ekaterina Kalyasova, Koshcheeva Valeria, Anastasia Zhirova, Irina Karpeeva, Nikolay Ilin

**Affiliations:** 1 Department of Biochemistry and Biotechnology, N.I. Lobachevsky State University of Nizhny Novgorod, 603950 Nizhny Novgorod, Russia; jsin@inbox.ru (Y.S.); katelyn@bk.ru (E.K.); sanchgin@gmail.com (K.V.); zhirovaav@mail.ru (A.Z.); karpeeva98@yandex.ru (I.K.); 2 Earth’s Electromagnetic Environment Laboratory, Institute of Applied Physics of Russian Academy of Sciences, 603600 Nizhny Novgorod, Russia; nilyin@yandex.ru

**Keywords:** Schuman resonance, electromagnetic field, redox metabolism, catalase, calmodulin, *Triticum aestivum* L., *Pisum sativum* L.

## Abstract

The Schumann Resonances (ScR) are Extremely Low Frequency (ELF) electromagnetic resonances in the Earth-ionosphere cavity excited by global lightning discharges. ScR are the part of electromagnetic field (EMF) of Earth. The influence of ScR on biological systems is still insufficiently understood. The purpose of the study is to characterize the possible role of the plant cell redox metabolism regulating system in the Schumann Resonances EMF perception. Activity of catalase and superoxide dismutase, their isoenzyme structure, content of malondialdehyde, composition of polar lipids in leaf extracts of wheat and pea plants treated with short-time (30 min) and long-time (18 days) ELF EMF with a frequency of 7.8 Hz, 14.3 Hz, 20.8 Hz have been investigated. Short-time exposure ELF EMF caused more pronounced bio effects than long-time exposure. Wheat catalase turned out to be the most sensitive parameter to magnetic fields. It is assumed that the change in the activity of wheat catalase after a short-term ELF EMF may be associated with the ability of this enzyme to perceive the action of a weak EMF through calcium calmodulin and/or cryptochromic signaling systems.

## 1. Introduction

Redox metabolism is a part of cellular metabolism and describes a finely regulated network of redox reactions and their cellular products [1]. The intracellular redox states are determined by pyridine nucleotide redox systems and thiol/disulfide redox systems [2]. Both of these systems are closely related to the reactions of the generation and utilization of reactive oxygen species (ROS), such as superoxide, hydrogen peroxide, singlet oxygen, and the hydroxyl radical [3,4].

In plant cells, ROS, such as hydrogen peroxide (H_2_O_2_) and superoxide (O^2-^•), are formed at a low level in various metabolic processes and they are central components of the signal transduction cascade involved in plant adaptation to the changing environment. They play an important role, for example, in the regulation of Na^+^ transport or redox-active ascorbate, promoting polysaccharide metabolism, and cell wall loosening and elongation. ROS also modify the different proteins’ functional activities and impact the activity of numerous protein–phosphorylation pathways, as well as transcription factors and other regulatory proteins, by directly affecting the protein’s oxidation state, such as by oxidizing methionine/cysteine residues and changes in the structure and the function of a protein malate dehydrogenase with oxidative modifications. However, at a high level, ROS can damage nucleic acids, proteins and lipids and cause cell damage and death [5,6,7,8,9,10].

Malondialdehyde (MDA) is one of several low molecular weight end products resulting from the degradation of certain primary and secondary products of lipid peroxidation. MDA is a breakdown product of polyunsaturated fatty acids in membranes under stress. It is believed that the rate of lipid peroxidation in terms of MDA can be used as an indicator for assessing plant resistance to stress [11,12,13].

It has been shown that the excessive production of ROS increases peroxidation at the cellular level, and the rate of this increase depends on the plant species and the severity of stress [14].

Catalase (CAT) and superoxide dismutase (SOD) are key enzymes in the metabolism of hydrogen peroxide and the superoxide anion radical [15,16,17].

Catalases (1.11.1.6) dismutate H_2_O_2_ to H_2_O and O_2_: (2H_2_O_2_→2H_2_O + O_2_) in plants have mainly been associated with peroxisomes (and also glyoxysomes) where it functions chiefly to remove the H_2_O_2_ formed during photorespiration (or during β-oxidation of fatty acids in glyoxysomes). In spite of its restricted location, it may play a significant role in the defense against oxidative stress since H_2_O_2_ can readily diffuse across membranes [18,19,20,21].

Classification of plant catalases is based on expression properties and tissue specificity. Class I catalases, to a greater extent than other catalases, are involved in the removal of H_2_O_2_ formed during photorespiration. Class II catalases are also expressed in mature plants, but with the highest levels in vascular tissues. The third group is formed by catalases, which are very abundant in seeds and young seedlings, but which are almost absent at later stages of development (although they may reappear during aging, see below). It is believed that their function is to remove H_2_O_2_ formed during the degradation of fatty acids in glyoxisomes [18].

Until recently, it was believed that most plant species contain three catalase genes, but at the moment it has been shown that the number of catalase genes in higher plants ranges from 1 to 7. Products of different genes of this family can function as catalases of the same class. For example, in Arabidopsis it has been shown that class III catalase can turn on CAT1 and CAT2 when localized in glyoxysomes. On the other hand, while CAT3—a class II catalase—is predominantly expressed in vascular tissues, it is also expressed in mesophyll cells as CAT2—a class I catalase. So, the main factors determining the function of the products of these genes are the place and time of their occurrence, not biochemical differences [20,22].

The number and expression of different CAT isozymes change during plant development, target tissue/organ and under different environmental conditions [20]. Two isoforms—CAT-1 and CAT-2—have been isolated from the wheat germ [23]. For example, in the common wheat (*Triticum aestivum* L.) genome, ten TaCAT genes were identified, forming three homoeologous groups, while phylogenetic analysis grouped TaCAT into three classes that showed conservative functional specialization based on their tissue-specific expression [24]. In pea, catalase appears to be encoded by only one gene [25] but five distinct isoforms were identified using isoelectric focusing (pH 5–7), with all catalase isoforms containing the 57 kDa subunit but having a different charge [26].

Superoxide dismutase (SOD, EC. 1.15.1.11) plays a major role in defense against oxygen radical-mediated toxicity in plant organisms. SOD has been characterized to convert superoxide anion radicals into oxygen and hydrogen peroxide. There are several isoforms of superoxide dismutase in plant cells: Cu/Zn-SOD (Mr 30–33 kDa) is found in the cytosol, chloroplasts, intermembrane space of mitochondria, in peroxisomes; Mn-SOD (Mr 75–94 kDa)—in the mitochondrial matrix; Fe-SOD (Mr 36–48 kDa)—in chloroplasts, proplastids [27,28,29]. Wheat and pea plants contain all three types of isoforms of superoxide dismutase; some of them have equal electrophoretic mobility [30,31].

Environmental adversity such as drought, high or low temperature, flood, UV- and other electromagnetic radiation often leads to the increased generation of reduced oxygen species in cells and, consequently, SOD is suggested to play an important role in plant stress tolerance [32,33]. 

Low-frequency electromagnetic fields (ELF EMF) are one of the exogenous factors, the action of which is realized through the non-specific nature of the perception and development of the response of a living cell [34,35]. As one of the possible mechanisms for the perception of low-frequency EMF the process of free radical oxidation initiated by reactive oxygen species (ROS) is discussed [36]. It was demonstrated that EMF could modify the activities of antioxidant enzymes such as peroxidase, superoxide dismutase, and catalase and increase the activity of the free radical ions in plant cells [37,38,39,40]**.**

It was hypothesized that the mechanism of the biological realization of this natural phenomenon on living systems is based on a magnetically sensitive process of free radical recombination which leads to the formation of reactive oxygen species and the modification of plant cell redox metabolism [41]. There are a number of reports that magnetic fields had the opposite effect on SOD activity: suppression of SOD activity in corn plants after 100 and 200 mT EMF [42], activation of this enzyme in Amaranthus retroflexus seedlings under the action of a magnetic field with an induction of 80 and 100 µT [43], and no changes in SOD activity were observed in radish after exposure to EMF with an induction of 185–650 µT [44]. As for the peroxide-degrading enzymes catalase and peroxidases, reports of their activation after exposure to ELF EMF predominate [43,45,46]. If the action of a strong unfavorable factor induces a state of oxidative stress in the cell, SOD, CAT and peroxidases work as a single system; however, weak effects such as EMF can apparently cause their scattered response, which allows the cell to regulate not only cleavage, but also the accumulation of some ROS. So, the balance between SOD and CAT activities in cells is crucial for determining the steady-state level of superoxide radicals and hydrogen peroxide [47]. In this case, we can assume the inclusion of enzymes that utilize ROS in intracellular signal transmission about the impact of weak EMF. 

Another widely discussed process—a candidate for the role of the receptor mechanism for low-intensity EMF—is the modification of calcium membrane channels and oscillations of intracellular calcium [48], or a change in the mobility and activity of the calcium ions themselves [49,50]. Both hypotheses are able to explain only some aspects of the phenomenology of the registered magnetobiological effects; none of them is currently sufficient and universal for the confident prediction of the behavior of a living system under the action of low-intensity magnetic radiation on a living cell. In recent years, a close relationship between ROS and Ca^2+^ signaling has been discussed, which occurs mainly through ROS-dependent Ca^2+^ channels and Ca^2+^-activated NADPH oxidases localized in the cell membrane [51]. Studies indicate that an increase in cytosolic calcium boosts the generation of H_2_O_2_. There was the report that calmodulin (Ca^2+^-CaM), a ubiquitous calcium-binding protein, binds to and activates some plant catalases in the presence of calcium [52]. A similar Ca^2+^-CaM domain was identified for one CAT isoform in wheat [53]. These results provide evidence indicating that calcium has a function in regulating H_2_O_2_ homeostasis, which in turn influences redox signaling in response to environmental signals in plants and gives us reason to assume that Ca^2+^-CaM can down-regulate H_2_O_2_ levels in plants by stimulating the catalytic activity of plant catalase. 

The Schumann Resonances (ScR) are Extremely Low Frequency (ELF) electromagnetic resonances in the Earth-ionosphere cavity excited by global lightning discharges. This natural electromagnetic field has likely existed on the Earth ever since the Earth had an atmosphere and an ionosphere, hence surrounding us throughout our evolutionary history. The background field of Schumann resonances exists in the atmosphere all the time. The ScR spectrum varies in amplitude and frequency depending on time of day, season and relative location on Earth. However, these changes in peak frequencies of different modes is less than 1 Hz and the amplitude changes are in the pT range; therefore, the Schumann resonance can be considered quasi-stationary in time and space. ScR has the intensity of a few pT and main peaks of the resonant characteristics at approximately 7.8 Hz, 14.3 Hz, 20.8 Hz [54,55]. Its influence on biological systems is still insufficiently understood, mainly due to the low magnitude of these fields. Presently, this phenomenon is being actively studied.

There are only a few studies of the mechanisms of perception by plant cells of low-frequency magnetic fields with frequencies close to the characteristics of the Schumann resonance. Cakmak et al. [45] reported an activating effect of ELF magnetic field (16 Hz 7 mT) on superoxide dismutase and catalase with a constant content of the lipoperoxidation product (malondialdehyde, MDA). Recently, our group [56] showed a similar picture of the antioxidant system response of pea plants to a pulsed magnetic field with a frequency of 15 Hz and an intensity of 1.5 mT. So, we hypothesized that ELF EMF with frequencies and intensities close to ScR may be received by wheat and pea plantlets and the cell redox regulating system plays a key in this perception mechanism.

## 2. Results

### Catalase and Superoxide Dismutase Activity

**Figure 1:**
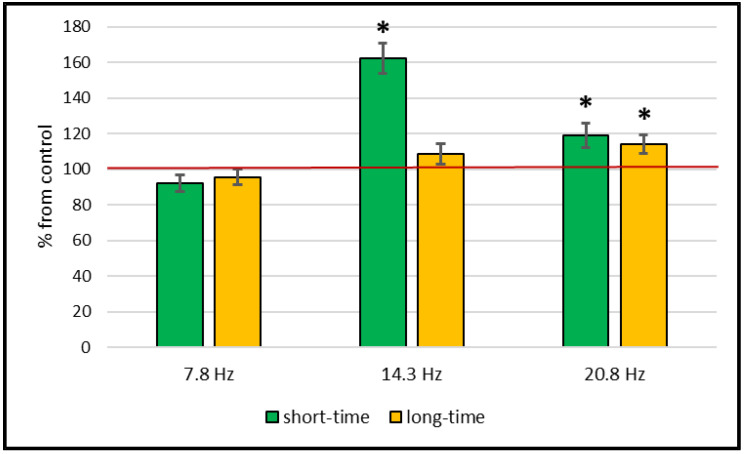
Effect of short-time (30 min) and long-time (18 days) treatment with magnetic fields of different frequencies on catalase activity in wheat plants. Data are represented as mean ± SD. *—a statistically significant difference from control (*p* ≤ 0.05); control level taken as 100%.

An increase in catalase activity after a short-time (30-min) exposure of wheat plants with a magnetic field with frequencies of 14.3 Hz and 20.8 Hz by 60% and 20% relative to the control level was shown. At the same time, catalase activity did not change after exposure to a magnetic field with a frequency of 7.8 Hz. However, during the 14-day cultivation of plants in a magnetic field (long-time exposure), catalase activity in wheat leaves changed only at a magnetic field frequency of 20.8 Hz—it increased by 18% (Figure 1, Table 1).

After a short-time exposure to magnetic fields of all studied frequencies on pea plants, catalase activity did not change (Table 1). After long-time exposure to a magnetic field, the activity of catalase in pea leaves changed only at a magnetic field frequency of 14.3 Hz—it decreased by 22%.

**Figure 2:**
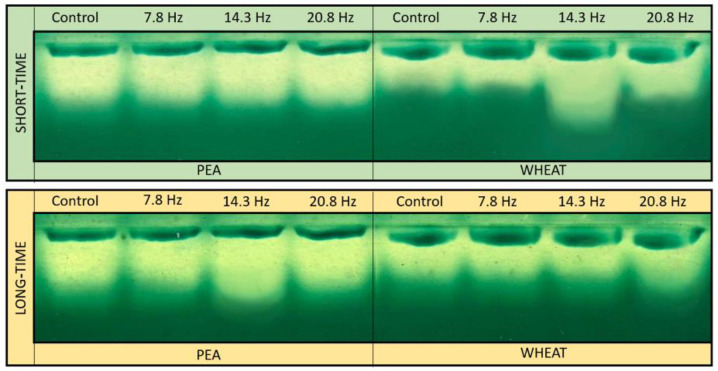
Enzyme activity patterns of catalase in extracts from wheat and pea leaves after ELF EMF influence.

In crude extracts of both plant species one CAT activity band was revealed by the in-gel staining assay (Figure 2). The dynamics of changes in the activity of catalase corresponded to the results of the assessment of the total activity of catalase (Table 1).

No change was found in the total activity of superoxide dismutase in wheat and pea plants after both short-time and long-time exposure (Table 1).

**Figure 3:**
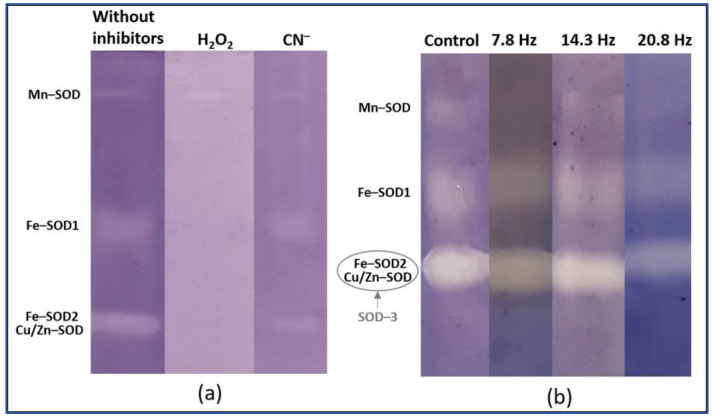
Isoenzyme activity patterns of SOD from wheat leaves: (**a**) inhibitory analysis of SOD; (**b**) typical pattern of the activity of SOD isoforms after exposure to ELF EMF of different frequencies.

SOD isoforms are identified by inhibitory analysis using H_2_O_2_ and CN**^−^** [57]. The inhibitory analysis of the SOD isoenzyme composition in wheat leaves revealed four isoforms, which were identified as Mn-SOD, Fe-SOD1, Fe-SOD2, and Cu/Zn-SOD (Figure 3a). The electrophoretic mobility of Fe-SOD2 and Cu/Zn-SOD was similar; therefore, their activity was further analyzed together as SOD-3 (Figure 3b). It was found that a short time of all MFs did not change the relative activity of all isoforms; however, after a long time of a magnetic field with frequencies of 7.8 Hz and 20.8 Hz, an increase in the activity of Mn-SOD was found (Table 2).

**Figure 4:**
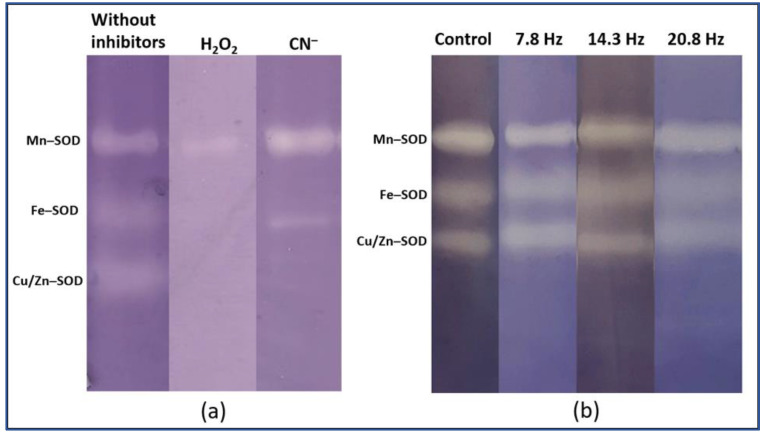
Isoenzyme activity patterns of SOD from pea leaves: (**a**) inhibitory analysis of SOD; (**b**) typical pattern of the activity of SOD isoforms after exposure to ELF EMF of different frequencies.

Electrophoregrams of crude extract of pea leaves revealed three bands with SOD activity, identified as Mn-SOD, Fe-SOD, and Cu/Zn-SOD (Figure 4). Neither a short-time nor a long-time of MF of all frequencies changed the relative activity of pea SOD isoforms.

**Figure 5:**
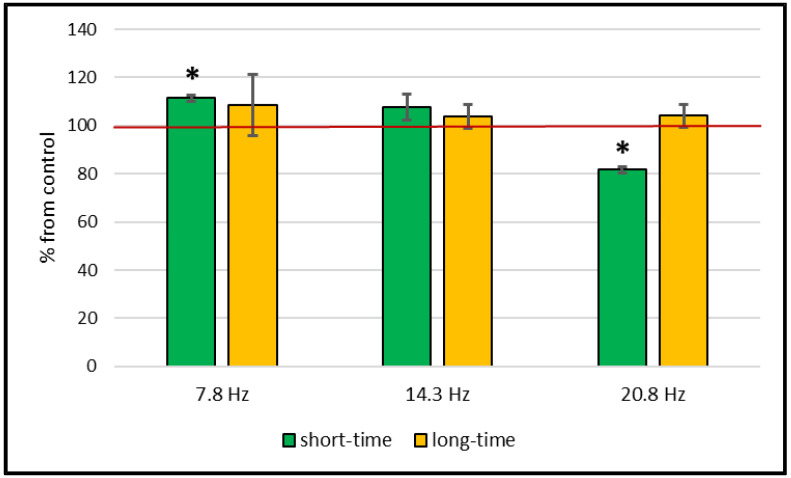
Effect of short-time (30 min) and long-time (18 days) ELF EMF of different frequencies on the content of malondialdehyde in wheat plants; Data are represented as mean ± SD. *—a statistically significant difference from control (*p* ≤ 0.05); control level taken as 100%.

An assessment of the content of the lipid peroxidation product—MDA—revealed its fluctuations only after a short-time exposure in wheat plants (Figure 5, Table 1). A magnetic field with a frequency of 7.8 Hz caused an increase in the MDA content by 11%, and a field with a frequency of 20.8 Hz caused a decrease by 16%.

**Figure 6:**
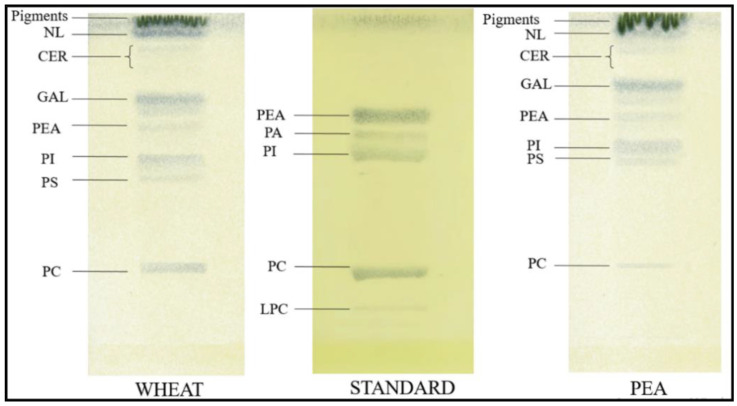
Typical thin layer chromatography (TLC) patterns from extracts of wheat and pea leaves.

Analysis of the composition of polar lipids in wheat and pea plants revealed the following groups: ceramides, galactolipids, phosphatidylethanolamine, phosphatidic acid, phosphatidylinositol, phosphatidylserine, phosphatidylcholine, lysophosphatidylcholine. The ratio of polar lipid groups was typical for plants [58]. No significant changes in the composition of lipid fractions were found after exposure to both short-term and long-term magnetic fields on wheat and pea plants (Figure 6).

Summarizing the above, wheat plants were more responsive to the effects of magnetic fields with Schumann resonance frequencies than peas. Among all the studied indicators, wheat catalase turned out to be the most sensitive to the effects of magnetic fields. Changes in its activity were registered both after a short-time and after a long-time of exposure. The total activity of SOD did not change after exposure to magnetic fields. At the same time, the activation of Mn-SOD was revealed, and only after a long-time of action of fields with frequencies of 7.8 Hz and 20.8 Hz. A short-time of exposure to magnetic fields with frequencies of 7.8 Hz and 20.8 Hz induced changes in the content of MDA, but a long time of exposure to fields of all frequencies did not cause differences from the control level. In general, a greater response was found from enzymes, associated directly with the processes of generation and utilization of ROS than with membrane lipids. For the development of the response, the type of treatment was more important than the frequency of the applied magnetic field. A short-time exposure time caused large changes in prooxidant and antioxidant activities.

Pea plants turned out to be almost insensitive to the action of the studied magnetic fields, only a decrease in catalase activity was found after a long-time of exposure to a magnetic field with a frequency of 14.3 Hz.

## 3. Discussion

The results obtained allow us to conclude that the Schumann resonance is not a stressor, since it does not cause a stable development of oxidative processes. Fluctuations in the content of MDA and enzyme activity were mainly observed after a short-time exposure but were absent after a long-time one. In the case of the stressful nature of the impact of the factor, we would observe a different picture: a gradual increase of oxidative processes with the depletion of the antioxidant reserves.

The two studied plant species showed a different sensitivity to the action of the studied fields. A more diverse response of wheat plants probably indicates the presence in these plants of a certain system of perception and signal transmission from the action of a magnetic field, which is partially or completely absent in pea plants. We suggest that this role can be played by the calcium signaling system, the level of intracellular calcium, calmodulin, and the enzymes regulated by it. For example, the TdCAT1 catalase isoform with a Ca^2+^-CaM domain was found in durum wheat. TdCAT1 protein shared high amino acid sequence identity with TaCAT1 from bread wheat (98.17% identity), OsCATC from rice (90.44% identity), HvCAT1 from barley (80% identity) and AtCAT2 from Arabidopsis (81.9% identity) [59]. The change in wheat catalase activity observed in our experiments after a short-time ELF EMF 14.3 and 20.8 Hz most likely depends on the direct work of the calcium-calmodulin system, which is able to transmit a signal about the effect of a magnetic field directly to the enzyme. In pea plants the Ca^2+^-CaM system is present but catalase activation did not occur. This is most likely due to the absence of a Ca^2+^-CaM domain in pea catalase.

In contrast to the multigene wheat catalase family [24,59], pea catalase appears to be encoded by only one gene [25] and its sequence is similar to cotton seed and maize catalase sequences [25,26]. It did not show homology to TdCAT1 durum catalase.

The perception of a magnetic field with the help of cryptochromes has been demonstrated for some animals [60]. At the same time, light-dependent inactivation was shown for the family of catalases homologous to wheat catalases. It is realized with the participation of cryptochromes [22]. Thus, a cryptochrome-dependent wheat catalase molecules modification may be another possible mechanism for ELF EMF perception and signal transmission.

The observed unique decrease in pea catalase activity after a long time of 14.3 Hz EMF could be due to post-translational modification [26]. Changes in catalase activity in wheat plants after a long time of EMT could be regulated both by post-translational modification of the enzyme and by changes in the expression of various catalase genes.

In general, the change in the content of MDA after a short-time ELF EMF corresponded to fluctuations in the activity of catalase in wheat plants. Thus, an increase in MDA content by 10% after EMF 7.8 Hz was accompanied by no changes in catalase activity, and after EMF 20.8 Hz a decrease in MDA content by 20% accompanied catalase activation. Most likely, this indicated the possibility of a direct effect of weak EMF on the lipid peroxidation processes, which proceed with the formation of a large number of radicals [41].

Superoxide dismutase plays a major role in defense against oxygen radical-mediated toxicity in aerobic organisms. In plants, many environmental adversities lead to the increased generation superoxide anion. SOD is immediately activated with an increase in the concentration of the superoxide radical and converts this ROS to less dangerous molecular oxygen and H_2_O_2_ [33].

There were no reports of a possible relationship between the calcium signaling sys-tem and the activity of the SOD isoforms for any plant cells. In our experiments, the absence of changes in the total SOD activity after any exposure to ELF EMF probably indicates that there was no direct effect of EMF on this family of enzymes and no increase in superoxide radical generation in different plant cell compartments. A slight increase in the relative activity of Mn-SOD in extracts obtained from wheat plants grown under long-time EMF conditions should be noted. Mn-SOD is predominantly concentrated in the mitochondria of plant cells (Srivalli 2001), so its activation may be associated with an increase in the flow of electrons to oxygen at the mitochondrial electron transport chain. Since no accumulation of lipid peroxidation products was observed in this case, the increase in Mn-SOD activity can probably be interpreted as an increase in the electron flow rate in the inner mitochondrial membrane. A similar increase in electron flow rate in photosynthetic membranes has been demonstrated by our group previously in wheat plants exposed to similar magnetic fields as the Schumann resonance [61].

Thus, the Schumann resonance—a background physical factor of the Earth—did not cause a pronounced systemic response from the studied components of plant redox metabolism. Significant changes in the activity of wheat catalase after a short-time of ELF EMF may be associated with the ability of this enzyme to perceive the action of a weak EMF through the Ca^2+^-CaM and/or cryptochromic signaling systems. These systems are involved in the implementation of fast signals but are little involved in the development of plant responses to long-term monotonous ELF EMF exposure. This explains the weak response of the components of the redox metabolism of wheat and pea plants when exposed to EMF for a long time.

## 4. Materials and Methods

### 4.1. Plant Growth and Magnetic Field Condition

Studies were carried out on soft spring wheat (*Triticum aestivum* L.) cv. Zlata, and pea (*Pisum sativum* L.) cv. Albumen. The seeds of wheat and pea plants were disinfected in a soapy solution, rinsed with clean water, and placed in containers on filter paper moistened with water. Further, germinated seeds were planted in containers with peat soil (composition, mg/L: N 100; P 80; K 130; pH 6.0). The plants were cultivated in a climate-controlled room at 23 °C with a 16-h light/8-h dark cycle, light intensity 2300–2500 lux and regular watering until the soil was saturated with water. In long-time exposure, the seeds of the experimental group were placed in a magnetic field, where they were during vegetation until the elimination of the experiment. In the case of a short-time exposure, the plants were cultivated outside the magnetic field until the age of 18 days, after which the plants of the experimental group were treated with a magnetic field once for 30 min, the control plants were under similar conditions, but without exposure to a magnetic field. To create an alternating magnetic field (frequency: 7.8 Hz or 14.3 Hz or 20.8 Hz, intensity 18 μT), two coaxially located Helmholtz rings on a wooden frame were used. A uniform magnetic field with a diameter of about 20 cm was located in the center between the rings. Plants of the control group were kept in identical conditions on a similar wooden structure but without Helmholtz rings. Plants were cultivated until 18 days from the moment of soaking. Studies were conducted on second leaves of wheat seedlings and on 3–4 tiers of leaves, counting from the top of pea plants.

### 4.2. Determination of MDA Content

The amount of malondialdehyde (TBA-active products) was determined according to the method of Kumar and Knowles [62], with modifications. Total TBA-reacting substances were extracted from fresh leaves and were expressed as MDA equivalents. 0.75 g of leaves was extracted with 5 mL of 0.1 M Tris-HCl buffer with 0.35 M NaCl. The crude extracts were strained through a layer of silk. Of each homogenate, 2.5 mL was vortexed with 2.5 mL of 20% (*w*/*v*) TCA for 10 min and then centrifuged at 12,000× *g* for 5 min. A 0.8-mL aliquot of each supernatant was then vortexed with 0.8 mL of 20% (*w*/*v*) TCA containing 0.5% (*w*/*v*) TBA, and the resulting solution was heated for 30 min at 95 °C. The samples were cooled and nonspecific absorbance of the supernatants at 600 nm was subtracted from the absorbance at 532 nm. The MDA equivalent was calculated on the resulting difference using the extinction coefficient of 155 mM^−1^ cm^−1^. The MDA content was calculated in µmol MDA/g dry weight. To determine the dry weight of plants, the samples were dried twice for 2 h at a temperature of 85° with a break of 12 h.

### 4.3. Determination of Antioxidant Enzyme Activity

The determination of superoxide dismutase (SOD) activity was carried out according to Giannopolitis and Ries [63]. Then, 0.5 g sample was triturated with 5 mL of 60 mM K-phosphate buffer (pH 7.8) containing 0.1 mM EDTA 0.05% Triton-X-100. The homogenate was centrifuged for 20 min at 12,000× *g* at 4 °C. Next, 0.5 mL of the supernatant was added to 1.5 mL of the reaction mixture (60 mM K-phosphate buffer, pH 7.8; methionine 13 mM; nitroblue tetrazolium chloride (NBT) 63 μM; riboflavin 1.3 μM). The reaction took place for 10 min under light irradiation of 15 W with fluorescent lamps. The complete reaction mixture, incubated in the dark, served as a dark control. The complete mixture without enzyme, in which the maximum color developed in the light, served as a light control. The reaction was interrupted by turning off the light, placing the samples in the dark. The optical density of the solutions was determined on a spectrophotometer at wavelengths of 560 nm. At 50% inhibition of the assay reaction, the SOD activity equals 1 unit. The activity was recalculated per mg of protein. The catalase (CAT) activity was determined by following the enzymic degradation of H_2_O_2_ [64,65]. Enzyme extraction was carried out as for SOD. Then, 1.9 mL of 60 mM K-phosphate buffer (pH = 7.8) was added to 0.1 mL of the supernatant; the reaction was started by adding 1 mL of 10 mM H_2_O_2_. A decrease in optical density was recorded spectrophotometrically at wavelengths of 240 nm for 1 min. Catalase activity was calculated using a molar extinction coefficient of 40 M^−1^ cm^−1^ with restatement on 1 mg of protein. In all studies, protein was determined by a modified Lowry method [66] using bovine serum albumin as a standard.

### 4.4. Superoxide Dismutase Isoforms Analysis

SOD isoforms activity was carried out by electrophoresis under non-denaturing conditions in a polyacrylamide gel with a concentration of 30% according to the method of Davis [67] using a VE-10 electrophoresis chamber (Helicon, Russia). To isolate SOD, 0.3 g of plant material was triturated with 2.5 mL of extraction medium (100 mM K-Na-phosphate buffer, pH 7.2; 1 mM EDTA; Triton X-100). The homogenate was centrifuged for 20 min at 16,000× *g* at 4 °C. The supernatant was placed on ice. The samples were aligned to the protein content; it was determined spectrophotometrically at 280 nm. Samples with 10 μg of protein were added to the pockets formed in gel. The parameters of the applied voltage or current intensity were I = 25 mA, U = 100 V per gel 10 cm. The gels were kept for 20 min in a solution of NBT in a potassium phosphate buffer of 50 mM (pH 7.8) and illuminated with fluorescent lamps for 20 min until stripes appeared. The different types of SOD were differentiated by performing the activity staining in gels previously incubated for 20 min at 25 °C in 50 mM sodium phosphate buffer, pH 7.8, containing either 3 mM KCN or 5 mM H_2_O_2_. Cu/Zn SODs are inhibited by KCN and H_2_O_2_, Fe-SODs are resistant to CN^−^ but inactivated by H_2_O_2_, and Mn-SODs are resistant to both inhibitors [68]. The obtained gels were photographed, the images were inverted in color and used to assess the activities of SOD isoforms using the ImageJ software. The number of zones of superoxide dismutase activity and the relative activity of each of the zones were determined.

### 4.5. Catalase Isoforms Analysis

Catalase isozymes were separated by 7% on denaturing polyacrylamide gels at 4 °C Equal amounts of protein per lane was loaded for the crude extracts.

Gels were stained according to the Santa-Cruz D.M. method [57] with modifications. The method is based on the ability of catalase to decompose H_2_O_2_ to form H_2_O and molecular oxygen. Gels were rinsed in distilled water followed by incubation in 0.003% (*v*/*v*) H_2_O_2_ for 10 min and then stained in 1% ferric chloride and 1% potassium ferricyanide solution (equal volumes of 2% (*w*/*v*) solutions of each component were added sequentially [68]).

### 4.6. Thin Layer Chromatography

Phospholipids were extracted by the method of Folch et al. [69]. Phospholipid fractions were determined by thin layer chromatography (TLC). Silica gel 60 F254 plates on aluminum (Merck, Germany) were used for chromatography. Chromatography was carried out in the system methyl acetate/*n*-propanol/chloroform/methanol/0.25% KCl (25:25:25:10:9), in a tight housing with a grinded lid. Solvent chambers were issued for at least 5 h. The solvents were triturated in a freshly prepared mixture of chloroform and methanol (2:1).

Lipid extraction was carried out in a mixture at the rate of 20 parts of the extracting mixture per 1 part of the tissue. Mixtures of the mixture and were filtered through a fat-free paper filter into a test tube, and then centrifuged for 15 min at 1000× *g*. After that, 0.2% 0.1% NaCl (KCl) was added to the supernatant, changed and centrifuged for 10 min at 1000× *g*. The consumption phase was selected and further worked only with the lower phase. The test sample was applied to a plate with silica gel 60 F254 on aluminum at a distance of 1.5 cm from the edge of the edge. The plate was placed in a solvent effect chromatography chamber, dissolved in methyl acetate/*n*-propanol/chloroform/methanol/0.25% KCl (25:25:25:10:9). The chromatography process was completed when the solvent reached the top plate. The plate was dried in an oven, chromatograms were represented by phosphoromolybdic acid, lipids were identified using lipid markers [70].

### 4.7. Statistical Analysis

All of the experiments were carried out with at least three independent repetition using three samples, and all of the data are expressed as the mean values ± the standard deviation (SD). Statistical analysis was performed using the Student’s t-test, and the differences between the treatments were expressed as significant at a level of *p* ≤ 0.05.

## Figures and Tables

**Table 1:** Value of redox metabolism parameters of wheat and pea plants after short-time (30 min) and long-time (18 days) exposure to ELF EMF. Data are represented as mean ± SD.

Investigated Parameter	Object	Frequency	7.8 Hz	14.3 Hz	20.8 Hz
SHORT-TIME					
Catalase activity, µM H_2_O_2_/min×mg of protein	Wheat	control	17.84 ± 2.05	15.89 ± 1.1	23.44 ± 1.37
		MF	16.24 ± 1.02	26.09 ± 1.38 *	27.95 ± 2.49 *
	Pea	control	24.32 ± 2.72	18.4 ± 3.63	26.65 ± 0.71
		MF	25.73 ± 1.36	21.29 ± 1.89	23.61 ± 0.68
SOD activity, r.u/mg of protein	Wheat	control	1.1 ± 0.41	0.61 ± 0.15	2.45 ± 0.51
		MF	0.81 ± 0.14	0.51 ± 0.11	1.92 ± 0.27
	Pea	control	0.42 ± 0.05	1.25 ± 0.11	1.84 ± 0.08
		MF	0.44 ± 0.04	1.02 ± 0.14	1.87 ± 0.17
MDA, µM/g of dry weight	Wheat	control	110.4 ± 1.11	136.78 ± 9.25	102.05 ± 1.2
		MF	123.11 ± 1.42 *	147.27 ± 7.36	83.45 ± 1.31 *
	Pea	control	235.87 ± 30.12	215.05 ± 9.42	177.68 ± 11.92
		MF	275.83 ± 8.54	195.41 ± 13.92	227.91 ± 27.11
LONG-TIME					
Catalase activity, µM H_2_O_2_/min×mg of protein	Wheat	control	20.75 ± 1.03	19.24 ± 0.92	29.62 ± 1.69
		MF	19.85 ± 0.88	20.9 ± 1.06	33.8 ± 1.61 *
	Pea	control	14.68 ± 0.42	22.07 ± 0.86	27.98 ± 0.74
		MF	14.83 ± 0.51	17.56 ± 0.99 *	26.73 ± 1.49
SOD activity, r.u/mg of protein	Wheat	control	2.53 ± 0.36	5.24 ± 0.5	6.69 ± 0.86
		MF	3.19 ± 0.58	4.09 ± 0.25	4.59 ± 0.68
	Pea	control	5.77 ± 1.13	3.98 ± 0.55	6.99 ± 0.82
		MF	3.3 ± 1.13	4.03 ± 0.72	12.47 ± 5.97
MDA, µM/g of dry weight	Wheat	control	168.19 ± 12.26	133.75 ± 8.05	100.00 ± 5.67
		MF	182.69 ± 21.20	139.02 ± 6.55	104.06 ± 4.73
	Pea	control	224.10 ± 13.01	176.06 ± 16.38	271.11 ± 15.89
		MF	231.65 ± 16.10	202.20 ± 17.85	260.46 ± 16.23

*—a statistically significant difference from control (*p* ≤ 0.05).

**Table 2:** Relative activity of superoxide dismutase isoforms in wheat and peas after short-time (30 min) and long-time (18 days) exposure to ELF EMF. Data are represented as mean ± SD.

Object	Isoform	Control	7.8 Hz	14.3 Hz	20.8 Hz
SHORT-TIME					
Wheat	Mn-SOD	13.6 ± 1.3	9.8 ± 0.3	7.3 ± 1.2	8.2 ± 0.9
	Fe-SOD	45.8 ± 0.2	46.7 ± 0.2	47.4 ± 1.3	46.6 ± 2.1
	SOD-3	43.5.0 ± 1.1	43.4 ± 0.4	45.2 ± 0.1	45.2 ± 3.0
Pea	Mn-SOD	26.7 ± 3.0	24.9 ± 2.4	23.7 ± 1.2	27.4 ± 2.0
	Fe-SOD	44.0 ± 2.6	44.2 ± 2.0	40.3 ± 3.2	38.8 ± 2.1
	SOD-3	29.3 ± 0.5	30.9 ± 4.4	36.0 ± 1.9	33.8 ± 0.1
LONG-TIME					
Wheat	Mn-SOD	6.1 ± 1.1	8.8 ± 2.2 *	5.2 ± 0.7	11.3 ± 1.2 *
	Fe-SOD	41.1 ± 1.3	30.8 ± 4.7	38.3 ± 1.3	44.6 ± 3.5
	SOD-3	52.8 ± 1.2	60.3 ± 3.4	56.5 ± 1.5	44 ± 2.8
Pea	Mn-SOD	34.8 ± 3.2	27.7 ± 1.2	37.3 ± 3.6	39.1 ± 1.3
	Fe-SOD	34 ± 2.8	36.4 ± 1.6	36.5 ± 0.9	30.8 ± 1.4
	SOD-3	31.2 ± 1.2	35.9 ± 0.8	26.2 ± 2.8	30 ± 1.2

*—a statistically significant difference from control (*p* ≤ 0.05).

## Data Availability

The data presented in this study are available on request from the corresponding author.
